# Supplementary material for: Measurements of the gingival papillae architecture using cone-beam computed tomography in young Chinese adults
Source: PeerJ. 2020 Sep 23;8:e10006. doi: 10.7717/peerj.10006 (PMC7519718; doi:10.7717/peerj.10006)
Supplement: Supplemental Information 2 [file peerj-08-10006-s002.docx]

Code book

Sex：

Male：1 Female：2

13-12: papilla between teeth 13 and 12

12-11: papilla between teeth 12 and 11

11-21 papilla between teeth 11 and 21

21-22 papilla between teeth 21 and 22

22-23 papilla between teeth 22 and 23

PH: papilla height

FLT: facial-lingual thickness

IDD:interdental distance
